# Effects of Dietary Fiber Type on Growth Performance, Serum Parameters and Fecal Microbiota Composition in Weaned and Growing-Finishing Pigs

**DOI:** 10.3390/ani12121579

**Published:** 2022-06-19

**Authors:** Zhiqian Lv, Zeyu Zhang, Fenglai Wang, Jiyu Guo, Xiaogang Zhao, Jinbiao Zhao

**Affiliations:** 1 Animal Husbandry and Fisheries Research Center, Guangdong Haid Group Co., Ltd., Guangzhou 510000, China; lzq2434390936@163.com; 2 State Key Laboratory of Animal Nutrition, Ministry of Agriculture and Rural Affairs Feed Industry Centre, China Agricultural University, Beijing 100193, China; mafic2017zeyu@163.com (Z.Z.); wangfl@cau.edu.cn (F.W.); 3 Guangdong Haid Group Co., Ltd., Guangzhou 510000, China; guojy@haid.com.cn; 4 Guangdong Provincial Key Laboratory of Research on the Technology of Pig-Breeding and Pig-Disease Prevention, Guangzhou 510000, China; zhaoxg01@haid.com.cn; 5 Guangdong Haid Institute of Animal Husbandry and Veterinary Co., Ltd., Guangzhou 510000, China

**Keywords:** soluble dietary fiber, insoluble dietary fiber, growth performance, fecal microbiota, pig

## Abstract

**Simple Summary:**

Much evidence has indicated that dietary fiber plays an important role in regulating performance and intestinal health of the pigs. Dietary fiber is divided into soluble (SDF) and insoluble dietary fiber (IDF) based on solubility. SDF is easier to be fermented by gut microbiota to produce more volatile fatty acids (VFA) than IDF, and IDF is beneficial to development of the gastrointestinal tract and microbial diversity. Until now, effects of different SDF to IDF ratios on pig performance and their microbial community have not been clear. To provide a scientifically recommended requirement of fiber types for pig nutrition, this study was conducted to explore the roles of different SDF to IDF ratios in weaned and growing-finishing pigs.

**Abstract:**

The objective of this study was to evaluate the effects of different SDF to IDF ratios on growth performance, serum indexes and fecal microbial community in pigs. Weaned and growing-finishing pigs were fed a diet containing five different ratios of SDF to IDF from 1:5 to 1:9 and from 1:3 to 1:7, respectively. Results showed a linear tendency that average daily gain (ADG) of weaned pigs decreased but the feed intake to weight gain ratio (F/G) increased as the ratio of SDF to IDF increased from 1:5 to 1:9 (*p* = 0.06). The ADG of growing-finishing pigs showed quadratic changes (*p* < 0.05) as ratios of SDF to IDF increased from 1:3 to 1:7. The Shannon index of fecal microbial diversity increased first and then decreased as the SDF to IDF ratio increased from 1:5 to 1:9 (*p* < 0.05). The Shannon and Chao indexes of fecal microbial diversity in growing-finishing pigs showed significant incremental linearly as the SDF to IDF ratio increased from 1:3 to 1:7 (*p* < 0.05). In conclusion, the recommended inclusion ratios of SDF to IDF in weaned and growing-finishing pigs diets are 1:7 and 1:5.

## 1. Introduction

Traditionally, dietary fibers are considered as anti-nutritional factors that decrease pig performance and nutrient digestibility because pigs cannot secret endogenous fiber-degrading enzymes to digest fiber components. However, dietary fibers are fermented by gut microbiota to produce lactic acid and volatile fatty acids (VFA), including mainly acetic acid, propionic acid and butyric acid [1]. Dietary fibers play a vital role in promoting development and physiological function of the gastrointestinal tract, such as intestinal peristalsis [2,3]. VFA, especially butyric acid, provide energy for epithelial cells and activate G protein-coupled receptors (GPR) and suppress histone deacetylase (HDAC) as active signaling molecules to improve immune function and intestinal integrity of the host [4]. A previous study reported that dietary supplementation of 5% wheat bran or corn bran was beneficial to pig performance, immune function and gut microbial composition [5]. However, Yu et al. reported that dietary supplementation of 10% wheat bran or 5% soybean hulls had no beneficial effects on growth performance and microbial community [6]. Therefore, different dietary fiber sources showed varying effects on pig performance and microbial community.

Dietary fibers are usually studied in pig nutrition based on chemical analysis of neutral detergent fiber (NDF) and acid detergent fiber (ADF), but the analyzed methods of NDF and ADF neglect soluble fibers. Recently, insoluble (IDF) and soluble dietary fiber (SDF) based on the physical properties of solubility have been used to evaluate roles of dietary fibers in regulating pig nutrition accurately [7]. Therefore, it is more reasonable and scientific to study dietary fibers using chemical components of IDF and SDF compared with NDF and ADF [8]. Corn bran and palm kernel meal contain more than 90% IDF as total dietary fiber (TDF); IDF is not easily fermented by gut microbiota and stimulates development and peristalsis of the intestine [9]. The proportion of SDF in sugar beet pulp is about 40% of the TDF. SDF is mostly degraded by microbiota and increasing the viscosity of the intestinal digesta, resulting in more VFA production and prolonging theretention period of the digesta [10]. Many studies have reported effects of dietary fiber levels on pig performance, nutrient digestibility and microbial composition [11,12,13], however, there are few studies focusing on effects of different SDF to IDF ratios on pig nutrition. We hypothesized that fiber types with different SDF to IDF ratios would have varying responses on pig performance. Therefore, the objective of this study was to explore the effects of different SDF to IDF ratios on growth performance, serum parameters, fecal microbial communities and their metabolites in weaned and growing-finishing pigs.

## 2. Materials and Methods

All protocols in the study followed animal care rules and were approved by the institution of China Agricultural University Animal Care and Use Ethics Committee (AW20602202-1-4). The experimental regulations and methods were approved and then performed according to the relevant criteria. Corn bran, palm kernel meal and sugar beet pulp were used to adjust dietary SDF to IDF ratios. The fiber composition and other chemical constituents are presented in the Table 1.

### 2.1. Animals, Diets and Experimental Design

A total of 240 weaned pigs (Duroc × Landrace × Yorkshire) with initial body weight (BW) of 7.98 ± 0.47 kg and age of 28 ± 1 d were allocated into five groups and fed diets with different ratio of soluble dietary fiber (SDF) to insoluble dietary fiber (IDF), which were 1:5, 1:6, 1:7, 1:8 and 1:9, respectively (Table 2). Each dietary treatment included six replicates and eight weaned pigs per replicate. The weaned pigs feeding trial lasted 28 d. A total of 180 growing-finishing pigs (Duroc × Landrace × Yorkshire) with initial body weight (BW) of 48.28 ± 1.07 kg and age of 90 ± 3 d were allocated into five groups and fed the diets with different ratios of SDF to IDF, which were 1:3, 1:4, 1:5, 1:6 and 1:7, respectively (Table 3). The growing-finishing pigs feeding trial lasted 56 d. The mixed premix of trace minerals and vitamins were provided for weaned and growing-finishing pigs to meet nutrient recommended requirement of pigs according to the guidelines of the NRC (2012) [14].

### 2.2. Feeding and Management

In both weaned and growing-finishing pig trials, pigs were provided ad libitum access to water and diets. Weaned pigs were housed in a pen (1.5 m × 1.2 m × 0.8 m). In the feeding room of weaned pigs, the humidity and temperature were controlled at 50~60% and 25 °C~28 °C, respectively. On days 0, 14 and 28, pig BW and feed consumed were weighed to determine the average daily feed intake (ADFI) and average daily gain (ADG). Growing-finishing pigs were housed in a pen (1.8 m × 1.5 m × 1.2 m). In the feeding room of the growing-finishing pigs, the humidity and temperature were controlled at 50~60% and 18 °C~20 °C, respectively. On days 0, 28 and 56, pig BW and feed consumed were weighed to evaluate pig ADFI and ADG. The feed conversion ratio (F/G) of weaned and growing-finishing pigs were calculated as the ratio of ADFI to ADG.

### 2.3. Sample Collection

At the end of the weaned and growing-finishing pigs feeding trials, blood samples (about 8 mL) from the weaned and growing-finishing pigs in each pen were collected into vacutainer tubes via jugular vena puncture. Then, about 1 mL of serum was collected after centrifuging at 3000× *g* for 15 min at 4 °C, and stored at −20 °C for further analysis. In addition, fecal samples of weaned and growing-finishing pigs in each pen were collected into 50 mL of centrifuge tubes using rectal stimulation and then stored at −80 °C before analysis of microbial community and volatile fatty acids (VFA).

### 2.4. Lab Analysis

#### 2.4.1. Chemical Composition

According to Official Methods of Analysis of AOAC international [15], feed ingredients were analyzed in terms of crude protein (CP; method 976.05), dry matter (DM; method 930.15), ether extract (EE; method 920.39), SDF and IDF (method 991.43). Total dietary fiber (TDF) was calculated as the sum of SDF and IDF. The NDF and ADF were determined using a fiber analyzer (Ankom Technology, Macedon, NY, USA). Gross energy (GE) was determined by an automatic adiabatic oxygen bomb calorimeter (Parr 1281, Automatic Energy Analyzer; Moline, IL, USA). All chemical compositions were analyzed in duplicate

#### 2.4.2. Serum Parameters

Serum parameters, including antioxidant capacity and immune function, were analyzed in duplicate according to the manufacturer’s instruction. Assay kits (Beijing Kangjia Bioengineering Company, Beijing, China) were used to analyze antioxidant capacity, including serum malondialdehyde (MDA), superoxide dismutase (SOD), glutathione peroxidase (GSH-Px) and total antioxidant capacity (T-AOC). Immunoglobulin G (IgG), Immunoglobulin A (IgA) and Immunoglobulin M (IgM) were determined using an Immunoglobulin Kit (Huaying Biotechnology Institute, Beijing, China).

#### 2.4.3. Fecal Microbiota Community

A Kit (Omega Bio-tek, Norcross, GA, USA) was applied to extract microbial DNA as described by Zhao et al. [5]. The genes of bacterial 16S ribosomal RNA in the V4-V5 variable region were amplified using PCR with primers. Integrity of PCR amplicons were analyzed by electrophoresis using a Tapestation Instruction (Agilent Technologies, Santa Clara, CA, USA). The PCR amplicons were extracted and purified by a DNA Gel Extraction Kit using 2% agarose gels (Axygen Biosciences, Union City, CA, USA). The output was quantified using QuantiFluor™ -ST and sequenced on an Illumina MiSeq system. QIIME software was used to demultiplex and quality-filtered raw Illumina fastq files. RDP database (http://rdp.cme.msu.edu/; accessed on 10 September 2020) was used to take the taxonomy-based analysis for operational taxonomic units (OTU) using an RDP classifier at a 90% confidence level. Alpha diversity (α-diversity) is defined as the mean diversity of microbial species, including Chao and Shannon indexes. Chao is an estimator based on abundance; thus, it requires data that refer to the abundance of individual samples belonging to a certain class. Simpson index is the measure of the degree of concentration when individuals are classified into types, equal to the probability of the two entities taken at random from the dataset of interest representing the same type.

#### 2.4.4. Fecal VFA Concentration

Concentrations of VFA, including acetate, propionate and butyrate, in the fecal samples were analyzed using methods described by a previous report [16]. Briefly, fecal samples (about 1 g) were placed into centrifuge tubes (10 mL) and 0.10% hydrochloric acid (2.0 mL). Tubes were placed in an ice bath for 25 min, then mixed and centrifuged at 15,000 rpm to harvest supernatant. Supernatant was filtered through a 0.45 μm nylon membrane filter (Millipore, Bedford, OH, USA) and then analyzed using a gas chromatograph system (Agilent HP 6890 Series, Santa Clara, CA, USA). The sample peaks were identified by comparing their retention times with internal standards of acetate, propionate, butyrate.

### 2.5. Statistical Analysis

A general linear model (GLM) of SAS was used to analyze the observations. Each pen was used as an experimental unit. Standardized operational taxonomic units (OTU) reads were applied to analyze bacterial microbial diversity, and composition was analyzed using standardized OTU reads according to a procedure in R software. The population of the microbial community in fecal samples of pigs at the phyla and genera levels were analyzed using Kruskal-Wallis analysis. The abundances of different bacteria were classified using the linear discriminant analysis (LDA) effect size algorithm if the logarithmic LDA values of bacteria exceeded 2.0. Differences were considered significant if *p* < 0.05, and a tendency if 0.05 < *p* < 0.10.

## 3. Results

### 3.1. Effects of Different SDF to IDF Ratio on Growth Performance of Weaned and Growing-Finishing Pigs

Effects of different SDF to IDF ratio on weaned pig performance are shown in Table 4. Weaned pigs in the dietary group with a 1:9 of SDF to IDF ratio had lower ADG at days 14–28 post-weaning than those with the other diets (*p* < 0.05). No significant differences in ADFI and F/G of weaned pigs at days 0~14 and days 14~28 were observed among dietary treatments with different ratio of SDF to IDF. The dietary group with a 1:7 of SDF to IDF ratio significant increased weaned pig ADG at days 14~28 (*p* < 0.05). The ADG of pigs at days 14–28 linearly decreased (*p* < 0.05) but F/G linearly increased (*p* < 0.05) as the ratio of SDF to IDF changed from 1:5 to 1:9. It was tendency that the ADG of pigs decreased (*p* = 0.06) and the F/G of pigs increased (*p* = 0.09) at days 0–28 as the ratio of SDF to IDF increased from 1:5 to 1:9.

Effects of different SDF to IDF ratio on growing-finishing pig performance are shown in Table 5. The pigs in the dietary group with 1:5 of SDF to IDF ratio showed greatest ADG at days 28–56 and a tendency to increase pig ADG at days 0–56 compared with those in the other treatments (*p* < 0.05). No significant differences in ADFI, ADG and F/G of weaned pigs at days 0–28 and days 28–56 were observed among dietary treatments with different ratio of SDF to IDF. The ADG of pigs at days 28–56 and days 0–56 quadratically changed (*p* < 0.05) as the ratio of SDF to IDF increased from 1:3 to 1:7.

### 3.2. Effects of Different SDF to IDF Ratio on Serum Immune and Antioxidance of Weaned and Growing-Finishing Pigs

Effects of different SDF to IDF ratio on weaned pig performance are shown in Table 6. There were no significant differences in serum concentrations of lg M, lg A, lg G and MDA, and activity of SOD, GSH-Px and T-AOC among all dietary treatments containing different ratio of SDF to IDF. In addition, it was a quadratic tendency that weaned pigs in the dietary group with 1:7 and 1:8 of SDF to IDF ratio had greater activity of T-AOC as the ratio of SDF to IDF increased from 1:5 to 1:9 (*p* = 0.08).

Effects of different SDF to IDF ratio on growing-finishing pig performance are shown in Table 7. There were no significant differences in serum concentrations of lg M, lg A and lg G, and activity of SOD, GSH-Px and T-AOC among all dietary treatments containing different ratio of SDF to IDF. In addition, the pigs in the dietary group with 1:3 and 1:5 of SDF to IDF ratio had lower serum concentration of MDA compared with those fed the other diets (*p* < 0.05). It was quadratic tendency that growing-finishing pigs had greater activity of GSH-Px as the ratio of SDF to IDF increased from 1:3 to 1:7 (*p* = 0.07); the groups with 1:5 and 1:6 of SDF to IDF ratio showed the greater activity of GSH-Px.

### 3.3. Effects of Different SDF to IDF Ratio on Fecal Microbiota Community of Weaned and Growing-Finishing Pigs

Effects of different SDF to IDF ratios on microbial diversity in feces of weaned and growing-finishing pigs ae shown in Table 8 and Table 9. The Shannon index of fecal microbial diversity in weaned pigs was significantly influenced by different SDF to IDF ratios (*p* < 0.05). The Shannon index of fecal microbial diversity increased first and then decreased as the SDF to IDF ratio increased from 1:5 to 1:9 (*p* < 0.05). The Shannon and Chao indexes of fecal microbial diversity in growing-finishing pigs were significantly affected by different SDF to IDF ratios (*p* < 0.05), which showed significant increment linearly as the SDF to IDF ratio increased from 1:3 to 1:7 (*p* < 0.05).

**Figure 1:**
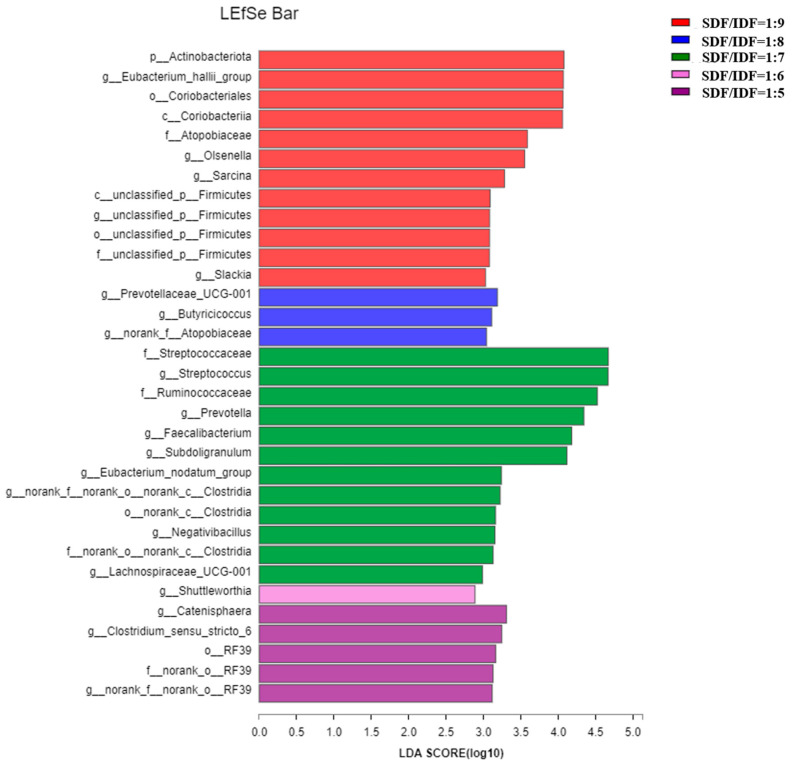
Effects of SDF to IDF ratio on fecal microbiota of weaned pigs. The abundances of different bacteria were classified using the linear discriminant analysis (LDA) effect size algorithm if the logarithmic LDA values of bacteria exceeded 2.0. Differences were considered significant if *p* < 0.05. *n* = 6. SDF, soluble dietary fiber; IDF, insoluble dietary fiber.

**Figure 2:**
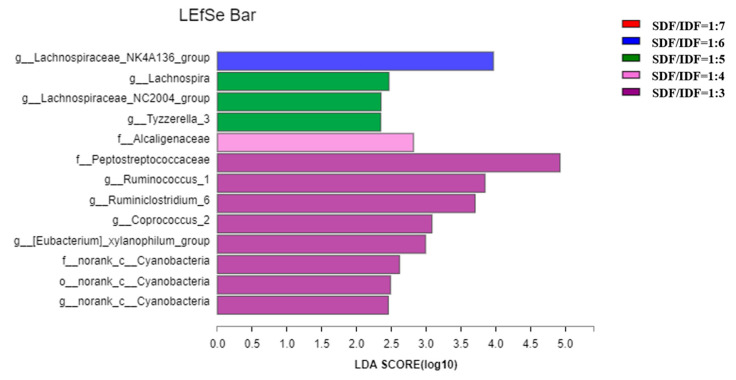
Effects of SDF to IDF ratio on fecal microbiota of growing-finishing pigs. The abundances of different bacteria were classified using the linear discriminant analysis (LDA) effect size algorithm if the logarithmic LDA values of bacteria exceeded 2.0. Differences were considered significant if *p* < 0.05. *n* = 6. SDF, soluble dietary fiber; IDF, insoluble dietary fiber.

Effects of different SDF to IDF ratios on different bacteria in feces of weaned and growing-finishing pigs are shown in Figure 1 and Figure 2. Dietary treatment with an SDF to IDF ratio of 1:7 significantly increased abundances of Streptococcaceae, Prevotella, Faecailbacterium and Clostridia in feces of weaned pigs compared with the other treatments (*p* < 0.05). Dietary treatment with a nSDF to IDF ratio of 1:3 significantly increased the population of Ruminiococcus_1 in feces of growing-finishing pigs compared with the other treatments (*p* < 0.05).

### 3.4. Effects of Different SDF to IDF Ratio on Fecal VFA Concentration of Weaned and Growing-Finishing Pigs

Results for effects of different SDF to IDF ratio on fecal VFA concentration in weaned and growing-finishing pigs are shown in Table 10 and Table 11. Fecal butyrate concentration in weaned pigs and all types of VFA in growing-finishing pigs were influenced by different dietary SDF to IDF ratio (*p* < 0.05). It was a tendency that fecal propionate concentration in weaned pigs was affected by different dietary SDF to IDF ratio (*p* = 0.06). In addition, concentrations of acetate, propionate, butyrate and total VFA in feces of weaned pigs and growing-finishing pigs showed linearly increases as the ratio of SDF to IDF increased from 1:3 to 1:7 (*p* < 0.05).

## 4. Discussion

### 4.1. Effects of Different SDF/IDF on Growth Performance

There are varying responses of fiber sources on growth performance of pigs due to their different physicochemical characteristics, such as fiber solubility and viscosity. Normally, wheat bran and alfalfa meal contain more IDF, but sugar beet pulp and pearled barley have a large amount of SDF [17,18,19]. Many publications reported that inclusion of wheat bran and sugar beet pulp had no significant effects on the ADFI in growing pigs [17,20]. However, a diet containing 50% alfalfa meal, or 30% sugar beet pulp decreased ADG and feed to gain ratio in growing-finishing pigs [21,22]. Moreover, Hopwood et al. reported inclusion of pearled barley, which contains a great quantity of SDF, decreased ADFI of weaned pigs by increasing digesta viscosity and prolonging satiety time [23]. A previous study reported moderate level of insoluble fiber sources, wheat bran and corn bran, were beneficial for growth performance of pigs during the first 2 weeks after weanling [5,24]. This positive effect on growth performance of piglets during the first 2 weeks might be because arabinoxylan in CB and WB enhanced the barrier function and health of the gut in piglets by regulating the production of butyrate. However, Yu et al. reported that a diet supplemented with 5% SB had negative effects on growth performance for weaned pigs on account of the concentration of anti-nutritional factors, especially trypsin inhibitors and α-galactosides [6]. Therefore, different fiber sources exert various effects on growth performance of pigs depending on fiber composition and physical characteristics. With an equal TDF level, the ADG of weaned pigs at days 14–28 linearly decreased but F/G linearly increased as the ratio of SDF to IDF ranged from 1:5 to 1:9, and the ADG of growing-finishing pigs at days 0~56 quadratically changed as the ratio of SDF to IDF increased from 1:3 to 1:7. Our findings indicate that growing-finishing pigs have a greater ability to utilize the SDF than weaned pigs due to their mature gastrointestinal tract. The recommended inclusion ratio of SDF to IDF in weaned pigs and growing-finishing pigs diets are 1:7 and 1:5, respectively. However, the effects of fiber physical characteristics on growth performance of pigs were not studied, such as particle size of the fiber and type of glycosidic bond.

### 4.2. Effects of Different SDF/IDF on Serum Immune and Antioxidant Capacity

Some publications have shown that VFA, especially butyrate, reduce the release of pro-inflammatory cytokines by inhibiting HDAC activity and activating the activator protein 1 (AP-1) signaling pathway in intestinal epithelial cells to improve the intestinal immune function of host [25,26]. Meanwhile, butyrate regulates the function of T lymphocytes through its receptor of GPR43 to reduce the level of inflammatory factor IL-2 and increase the secretion of anti-inflammatory factor interleukin-4 (IL-4) and antimicrobial peptide LL-37, resulting in an improvement of the host immune systems] [27,28]. In the study, concentrations of inflammatory cytokines were not analyzed, but different SDF to IDF ratios did not affect serum immunoglobulin concentrations in weaned pigs and growing-finishing pigs. This result indicates that more VFA production as the proportion of SDF increased cannot improve the serum immune function of pigs. The different finding in our study compared with the report mentioned above may be associated with self-regulation of the serum internal environment by the host. It is difficult to clarify the result of serum MDA levels in the different groups with different SDF/IDF ratios. The previous publication showed that specific gut microbiota were associated with fat metabolism and antioxidant function [29]. Therefore, serum MDA levels may be associated with some gut microbiota shaped by the specific SDF to IDF ratio. However, more studies to answer the irregular MDA levels should be explored.

### 4.3. Effects of Different SDF/IDF on Fecal Microbiota and Their Metabolites

There is a large variation in fermentability of dietary fiber in the hindgut of pigs, which ranges from 48% to 95% [10,30]. Apparent total tract digestibility (ATTD) of SDF was reported to be 20% greater than that of IDF, indicating that SDF is more fermentable by gut microbiota than IDF in the intestine of pigs [31,32]. Pectin and soluble hemicellulose are more easily fermented than cellulose, and β-glucan is almost completely fermented in the hindgut of pigs [30]. Zhao et al. found that ATTD of TDF in different ingredients fed to growing pigs was 37.78% for wheat bran, 71.87% for oat bran, 72.54% for sugar beet pulp and 72.31% for soybean hulls [33]. The poor digestibility of wheat bran can be ascribed to its high insoluble fiber content, which makes wheat bran less fermentable compared with sugar beet pulp and soybean hulls that contain highly fermentable pectin substances. In our study, concentrations of acetate, propionate, butyrate and total VFA in feces of weaned pigs and growing-finishing pigs linearly increased as the ratio of SDF to IDF increased, indicating that SDF is easier to ferment by gut microbiota than IDF to produce VFA, and there is no strong correlation between pig performance and VFA production.

The Shannon index of fecal microbial α-diversity increased first and then decreased as the SDF to IDF ratio increased from 1:5 to 1:9, and fecal microbial α-diversity in growing-finishing pigs showed a significant linear increment as the SDF to IDF ratio increased from 1:3 to 1:7. Our results are consistent with the previous report that IDF plays more important roles in promoting microbial growth than the SDF. However, there is a limitation on microbial fermentability of fiber components in weaned pigs due to their immature gastro-intestinal tract compared with the growing-finishing pigs, leading to the greatest fecal microbial α-diversity in the dietary group with an SDF/IDF ratio of 1:7. In addition, dietary treatment with an SDF to IDF ratio of 1:7 significant increased abundances of *Prevotella* and *Faecailbacterium* in feces of weaned pigs compared to the other treatments. *Prevotella* and *Faecailbacterium* are the primary bacteria for dietary fiber fermentation, and produce propionate and butyrate, respectively [34,35].

SDF and IDF can be provided by different sources of plant-derived feed ingredients, but they always have varying physical characteristics and natural internal structures of the fiber, resulting in different biological effects on pig performance and health. Therefore, limitations of this study include the role of molecular structure and physical characteristics of fiber components among feed ingredients in regulating growth performance and intestinal function, and these should be further explored with respect to particle size of the fiber and viscosity. In addition, although the suitable inclusion ratio of SDF to IDF in weaned pig and growing-finishing pig diets has been clarified, varying molecular mechanisms of SDF and IDF on pig performance are still unclear.

## 5. Conclusions

In conclusion, different dietary SDF to IDF ratios show different responses with respect to growth performance, fecal microbial community and VFA production. Growing-finishing pigs have a greater fiber-degrading capacity compared to weaned pigs. Although VFA concentrations increased gradually as dietary SDF to IDF ratios increased, the recommended inclusion ratio of SDF to IDF in weaned pigs and growing-finishing pigs diets are 1:7 and 1:5. However, the potential mechanisms of better pig performance after treatment of 1:7 and 1:5 of SDF to IDF in weaned and growing-finishing pigs is unclear, and the finding above could be associated with changes of physical characteristics and the microbial community. Further studies on interactions among fiber types, properties of intestinal digesta and gut microbiota should be carried out.

## Figures and Tables

**Table 1:** Chemical composition of feed ingredients used in the study.

Items	Corn	Soybean Meal	Corn Bran	Palm Kernel Meal	Sugar Beet Pulp
Total dietary fiber	9.1	22.2	53.1	49.0	69.0
Soluble dietary fiber	1.0	4.2	5.0	3.1	27.4
Insoluble dietary fiber	8.1	17.9	48.1	46.2	42.5
Neutral detergent fiber	8.2	11.1	44.0	44.8	37.7
Acid detergent fiber	2.1	6.2	14.2	24.6	21.2
Gross energy	15.9	17.0	17.3	17.9	16.2
Dry matter	85.2	87.4	92.1	90.0	86.9
Crude protein	7.7	42.1	14.8	15.6	8.7
Ether extract	2.5	1.4	3.9	5.6	0.4

**Table 2:** Ingredient composition and nutrient levels of weaned pig diets.

Items, %	SDF/IDF				
	1:5	1:6	1:7	1:8	1:9
Sugar beet pulp	14.00	8.00	5.00	2.00	0.00
Corn bran	3.00	12.00	14.00	16.70	18.00
Palm kernel meal	0.00	0.00	2.50	4.00	5.28
Corn starch	52.53	51.89	50.70	49.81	50.29
Whey powder	7.00	5.00	5.00	5.00	4.00
Soy protein concentrate	19.00	18.60	18.65	18.59	18.66
Dicalcium phosphate	1.60	1.65	1.50	1.40	1.35
Limestone	0.80	0.75	0.85	0.90	0.95
NaCl	0.40	0.40	0.40	0.40	0.40
L-Lysine-HCl	0.63	0.65	0.46	0.35	0.26
DL-Methionine	0.18	0.18	0.17	0.16	0.16
L-Threonine	0.30	0.32	0.23	0.17	0.14
L-Tryptophan	0.06	0.06	0.04	0.02	0.01
Premix ^1^	0.50	0.50	0.50	0.50	0.50
Total	100.00	100.00	100.00	100.00	100.00
**Nutritive levels, % ^2^**					
DE, Kcal/kg	3549	3546	3545	3540	3547
CP	15.33	15.30	15.33	15.30	15.32
SID Lys	1.35	1.35	1.35	1.35	1.35
SID Met	0.39	0.39	0.39	0.39	0.39
SID Thr	0.79	0.79	0.79	0.79	0.80
SID Trp	0.22	0.22	0.22	0.22	0.22
TDF	12.40	12.65	12.65	12.53	12.33
SDF	2.07	1.80	1.59	1.39	1.24
IDF	10.33	10.85	11.06	11.14	11.09

^1^ Premix provided the following per kg of complete diet for growing pigs: vitamin A, 12,000 IU; vitamin D3, 2500 IU; vitamin E, 30 IU; vitamin K3, 3.0 mg; vitamin B6, 3.0 mg; vitamin B12, 12 μg; riboflavin, 4.0 mg; thiamine, 1.5 mg; niacin, 40 mg; pantothenic acid, 15 mg; folacin, 0.7 mg; biotin, 44 μg; choline chloride, 400 mg; Cu, 10 mg; Fe, 90 mg; Zn, 80 mg; Mn, 30 mg; I, 0.35 mg; Se, 0.3 mg. ^2^ Calculated values. DE, digestible energy; CP, crude protein; SID, standardized ileal digestibility; TDF, total dietary fiber; SDF, soluble dietary fiber; IDF, insoluble dietary fiber; Lys, lysine; Met, Methionine; Thr, Threonine; Trp, Tryptophan.

**Table 3:** Ingredient composition and nutrient levels of growing-finishing pig diets.

Items, %	SDF/IDF				
	1:3	1:4	1:5	1:6	1:7
Corn	71.33	74.17	72.59	73.59	73.92
Soybean meal	7	9	10	10	10
Sugar beet pulp	9.5	5.2	3.7	2	1
Palm kernel meal	0	1.8	5	6	7
Soy protein concentrate	4.8	3.5	3	3	3
Soy oil	2.3	1.6	1.3	1.1	0.9
Dicalcium phosphate	1.65	1.5	1.4	1.4	1.4
Limestone	0.95	1	1.1	1.1	1.1
NaCl	0.3	0.3	0.3	0.3	0.3
L-Lysine-HCl	1.02	0.9	0.68	0.62	0.55
DL-Methionine	0.15	0.15	0.15	0.14	0.13
L-Threonine	0.4	0.3	0.22	0.2	0.16
L-Tryptophan	0.1	0.08	0.06	0.05	0.04
Premix ^1^	0.5	0.5	0.5	0.5	0.5
Total	100	100	100	100	100
**Nutritive levels, % ^2^**					
DE, Kcal/kg	3330	3331	3330	3335	3334
CP	14.80	14.73	14.76	14.75	14.70
SID Lys	1.34	1.34	1.35	1.36	1.36
SID Met	0.36	0.36	0.37	0.36	0.36
SID Thr	0.78	0.74	0.77	0.78	0.78
SID Trp	0.21	0.21	0.22	0.22	0.22
TDF	14.67	13.25	13.86	13.24	13.06
SDF	3.61	2.60	2.31	1.88	1.64
IDF	11.06	10.65	11.55	11.36	11.41

^1^ Premix provided the following per kg of complete diet: vitamin A, 5,600 IU; vitamin D3, 2,200 IU; vitamin E, 21.6 IU; vitamin K3, 1.8 mg; vitamin B6, 1.8 mg; vitamin B12, 12 μg; riboflavin, 4 mg; thiamine, 0.88 mg; niacin, 20 mg; pantothenic acid, 10 mg; folacin, 0.4 mg; biotin, 40 μg; choline chloride, 320 mg; Cu, 15 mg; Fe, 100 mg; Mn, 10 mg; I, 0.3 mg; Se, 0.3 mg. ^2^ Calculated values. DE, digestible energy; CP, crude protein; SID, standardized ileal digestibility; TDF, total dietary fiber; SDF, soluble dietary fiber; IDF, insoluble dietary fiber; Lys, lysine; Met, Methionine; Thr, Threonine; Trp, Tryptophan.

**Table 4:** Effects of SDF to IDF ratio on growth performance of weaned pigs.

Items	Different SDF/IDF Ratios					SEM	*p*-Value		
	1:5	1:6	1:7	1:8	1:9		Treatment	Linear	Quadratic
BW									
d 0 kg	7.95	7.97	7.99	8.03	7.98	0.47	0.99	0.93	0.94
d 14 kg	12.12	12.11	12.32	12.41	12.07	0.59	0.99	0.91	0.73
d 28 kg	19.32	19.28	19.63	18.91	18.14	0.82	0.75	0.31	0.47
d 0~14									
ADG g/d	299	296	309	314	293	13.43	0.77	0.90	0.38
ADFI g/d	442	416	444	442	430	15.23	0.71	0.96	0.99
F/G	1.48	1.41	1.46	1.41	1.47	0.05	0.77	0.89	0.36
d 14~28									
ADG g/d	515 ^a^	512 ^a^	522 ^a^	464 ^a,b^	434 ^b^	21.91	0.03	0.01	0.15
ADFI g/d	796	781	829	741	726	32.72	0.22	0.09	0.23
F/G	1.55	1.53	1.59	1.60	1.68	0.04	0.13	0.02	0.38
d 0~28									
ADG g/d	407	404	416	389	364	16.16	0.20	0.06	0.18
ADFI g/d	619	599	637	591	578	22.21	0.43	0.23	0.44
F/G	1.52	1.48	1.54	1.53	1.59	0.03	0.27	0.09	0.25

^a,b^ Means within a row with different letters differ among different dietary treatments (*p* < 0.05), *n* = 6. BW, body weight; ADFI, average daily feed intake; ADG, average daily gain; F/G, feed intake to weight gain ratio; SEM, standard error of the mean; SDF, soluble dietary fiber; IDF, insoluble dietary fiber.

**Table 5:** Effects of SDF to IDF ratio on growth performance of growing-finishing pigs.

Items	Different SDF/IDF Ratios					SEM	*p*-Value		
	1:3	1:4	1:5	1:6	1:7		Treatment	Linear	Quadratic
BW									
d 0 kg	48.16	48.33	48.49	48.32	48.05	1.65	0.99	0.97	0.90
d 28 kg	74.27	75.74	77.24	75.44	74.49	3.54	0.99	0.99	0.64
d 56 kg	98.73	101.79	104.07	100.61	99.10	5.34	0.92	0.98	0.39
d 0~28									
ADG g/d	933	979	1027	969	944	20.54	0.59	0.92	0.14
ADFI g/d	2477	2427	2537	2449	2414	37.54	0.97	0.82	0.75
F/G	2.65	2.47	2.47	2.54	2.55	0.07	0.34	0.51	0.09
d 28–56									
ADG g/d	873 ^b^	930 ^a,b^	959 ^a^	899 ^a,b^	879 ^b^	21.65	0.04	0.75	0.01
ADFI g/d	2956	3041	3059	2899	2857	38.86	0.59	0.31	0.28
F/G	3.39	3.28	3.20	3.23	3.25	0.08	0.85	0.42	0.45
d 0–56									
ADG g/d	903	955	993	934	912	24.65	0.08	0.95	0.01
ADFI g/d	2719	2737	2800	2676	2638	37.31	0.89	0.56	0.50
F/G	3.01	2.86	2.82	2.87	2.89	0.10	0.49	0.32	0.16

^a,b^ Means within a row with different letters differ among different dietary treatments (*p* < 0.05), *n* = 6. BW, body weight; ADFI, average daily feed intake; ADG, average daily gain; F/G, feed intake to weight gain ratio; SEM, standard error of the mean; SDF, soluble dietary fiber; IDF, insoluble dietary fiber.

**Table 6:** Effects of SDF to IDF ratio on serum immune and antioxidase activity of weaned pigs.

Items	Different SDF/IDF Ratios					SEM	*p*-Value		
	1:5	1:6	1:7	1:8	1:9		Treatment	Linear	Quadratic
IgM g/L	1.59	1.58	1.66	1.68	1.58	0.02	0.73	0.72	0.36
IgA g/L	2.52	2.48	2.52	2.49	2.59	0.02	0.94	0.62	0.61
IgG g/L	18.29	16.69	16.94	19.03	18.24	1.03	0.10	0.30	0.18
SOD U/mL	94.88	81.78	87.87	75.93	87.53	7.45	0.21	0.26	0.15
GSH-Px U/mL	309	314	331	310	317	16.07	0.20	0.50	0.28
T-AOC U/mL	7.54	7.70	8.54	8.68	7.63	0.32	0.13	0.51	0.08
MDA nmol/mL	5.61	5.08	5.27	5.31	5.79	0.18	0.52	0.39	0.41

*n* = 6. lg A, immunoglobulin A; lg M, immunoglobulin M; lg G, immunoglobulin G; MDA, malondialdehyde; SOD; superoxide dismutase; T-AOC, total antioxidant capacity; GSH-Px, glutathione peroxidase; SDF, soluble dietary fiber; IDF, insoluble dietary fiber.

**Table 7:** Effects of SDF to IDF ratio on serum immune and antioxidase activity of growing-finishing pigs.

Items	Different SDF/IDF Ratios					SEM	*p*-Value		
	1:3	1:4	1:5	1:6	1:7		Treatment	Linear	Quadratic
Ig M g/L	1.42	1.52	1.49	1.42	1.43	0.05	0.94	0.83	0.41
Ig A g/L	3.41	3.29	3.33	3.28	3.32	0.16	0.91	0.55	0.47
Ig G g/L	11.66	12.18	11.79	10.63	11.69	0.63	0.15	0.39	0.12
SOD U/mL	42.52	34.23	42.77	38.42	47.77	3.45	0.40	0.36	0.21
GSH-Px U/mL	197	195	225	229	201	13.34	0.21	0.50	0.07
T-AOC U/mL	6.36	5.24	6.28	5.58	6.62	0.38	0.18	0.55	0.12
MDA nmol/mL	3.65 ^b^	4.34 ^a^	3.66 ^b^	4.13 ^a,b^	3.89 ^a,b^	0.16	0.03	0.30	0.27

^a,b^ Means within a row with different letters differ among different dietary treatments (*p* < 0.05), *n* = 6. lg A, immunoglobulin A; lg M, immunoglobulin M; lg G, immunoglobulin G; MDA, malondialdehyde; SOD; superoxide dismutase; T-AOC, total antioxidant capacity; GSH-Px, glutathione peroxidase; SDF, soluble dietary fiber; IDF, insoluble dietary fiber.

**Table 8:** Effects of SDF to IDF ratio on fecal microbial diversity of weaned pigs.

Items	SDF/IDF					SEM	*p*-Value		
	1:5	1:6	1:7	1:8	1:9		One-Way	Linear	Quadratic
Shannon	3.68 ^a,b^	3.60 ^a,b^	4.01 ^a^	3.97 ^a^	3.46 ^b^	0.16	0.01	0.43	0.02
Chao	614	582	590	579	611	22.59	0.41	0.72	0.64

^a,b^ Means within a row with different letters differ among different dietary treatments (*p* < 0.05), *n* = 6. SDF, soluble dietary fiber; IDF, insoluble dietary fiber.

**Table 9:** Effects of SDF to IDF ratio on fecal microbial diversity of growing-finishing pigs.

Items	SDF/IDF					SEM	*p*-Value		
	1:3	1:4	1:5	1:6	1:7		One-Way	Linear	Quadratic
Shannon	2.80 ^b^	3.28 ^a,b^	3.41 ^a,b^	3.67 ^a^	3.55 ^a^	0.21	0.01	0.03	0.76
Chao	512 ^b^	501 ^b^	598 ^a^	570 ^a,b^	562 ^a,b^	22.59	0.01	0.02	0.39

^a,b^ Means within a row with different letters differ among different dietary treatments (*p* < 0.05), *n* = 6. SDF, soluble dietary fiber; IDF, insoluble dietary fiber.

**Table 10:** Effects of SDF to IDF ratio on fecal VFA (mg/g) concentration of weaned pigs.

Items	SDF/IDF					SEM	*p*-Value		
	1:5	1:6	1:7	1:8	1:9		One-Way	Linear	Quadratic
Acetate	5.25	5.00	4.60	4.65	4.32	0.54	0.21	0.01	0.45
Propionate	3.16	3.17	2.83	2.36	2.03	0.37	0.06	0.01	0.64
Butyrate	1.67 ^a^	1.57 ^a^	1.66 ^a^	1.19 ^a,b^	0.87 ^b^	0.15	0.01	0.01	0.56
Total VFA	10.08 ^a^	9.74 ^a^	9.09 ^a,b^	8.19 ^a,b^	7.22 ^b^	0.82	0.03	0.01	0.35

^a,b^ Means within a row with different letters differ among different dietary treatments *(p* < 0.05), *n* = 6. VFA, volatile fatty acids; SDF, soluble dietary fiber; IDF, insoluble dietary fiber.

**Table 11:** Effects of SDF to IDF ratio on fecal VFA (mg/g) concentration of growing-finishing pigs.

Items	SDF/IDF					SEM	*p*-Value		
	1:3	1:4	1:5	1:6	1:7		One-Way	Linear	Quadratic
Acetate	12.02 ^a^	11.25 ^a^	9.93 ^a,b^	9.49 ^a,b^	6.26 ^b^	1.23	0.01	0.01	0.43
Propionate	7.53 ^a,b^	8.99 ^a^	5.34 ^b,c^	4.79 ^b,c^	4.14 ^c^	0.87	0.01	0.01	0.52
Butyrate	6.31 ^a^	4.71 ^a^	4.28 ^b^	3.32 ^b,c^	1.80 ^c^	0.63	0.01	0.01	0.49
Total VFA	25.86 ^a^	24.96 ^a^	19.54 ^a,b^	17.60 ^b^	12.21 ^c^	1.59	0.01	0.01	0.65

^a,b,c^ Means within a row with different letters differ among different dietary treatments (*p* < 0.05), *n* = 6. VFA, volatile fatty acids; SDF, soluble dietary fiber; IDF, insoluble dietary fiber.

## Data Availability

The data that support the findings of this study are available from the corresponding author upon reasonable request.
